# Umbilical cord-mesenchymal stem cells induce a memory phenotype in CD4^+^ T cells

**DOI:** 10.3389/fimmu.2023.1128359

**Published:** 2023-06-20

**Authors:** Ezgi Sengun, Tim G. A. M. Wolfs, Valéry L. E. van Bruggen, Bram van Cranenbroek, Elles R. Simonetti, Daan Ophelders, Marien I. de Jonge, Irma Joosten, Renate G. van der Molen

**Affiliations:** ^1^ Department of Laboratory Medicine, Laboratory of Medical Immunology, Radboud University Medical Center Nijmegen, Radboud Institute for Molecular Life Sciences, Nijmegen, Netherlands; ^2^ Department of Pediatrics and GROW School for Oncology and Reproduction, Maastricht University Medical Center, Maastricht, Netherlands

**Keywords:** immunomodulation, umbilical cord mesenchymal stem cells, memory T cells, central memory, CD4 + T cells, cell contact, flow cytometry

## Abstract

Inflammation is a physiological state where immune cells evoke a response against detrimental insults. Finding a safe and effective treatment for inflammation associated diseases has been a challenge. In this regard, human mesenchymal stem cells (hMSC), exert immunomodulatory effects and have regenerative capacity making it a promising therapeutic option for resolution of acute and chronic inflammation. T cells play a critical role in inflammation and depending on their phenotype, they can stimulate or suppress inflammatory responses. However, the regulatory effects of hMSC on T cells and the underlying mechanisms are not fully elucidated. Most studies focused on activation, proliferation, and differentiation of T cells. Here, we further investigated memory formation and responsiveness of CD4^+^ T cells and their dynamics by immune-profiling and cytokine secretion analysis. Umbilical cord mesenchymal stem cells (UC-MSC) were co-cultured with either αCD3/CD28 beads, activated peripheral blood mononuclear cells (PBMC) or magnetically sorted CD4^+^ T cells. The mechanism of immune modulation of UC-MSC were investigated by comparing different modes of action; transwell, direct cell-cell contact, addition of UC-MSC conditioned medium or blockade of paracrine factor production by UC-MSC. We observed a differential effect of UC-MSC on CD4^+^ T cell activation and proliferation using PBMC or purified CD4^+^ T cell co-cultures. UC-MSC skewed the effector memory T cells into a central memory phenotype in both co-culture conditions. This effect on central memory formation was reversible, since UC-MSC primed central memory cells were still responsive after a second encounter with the same stimuli. The presence of both cell-cell contact and paracrine factors were necessary for the most pronounced immunomodulatory effect of UC-MSC on T cells. We found suggestive evidence for a partial role of IL-6 and TGFβ in the UC-MSC derived immunomodulatory function. Collectively, our data show that UC-MSCs clearly affect T cell activation, proliferation and maturation, depending on co-culture conditions for which both cell-cell contact and paracrine factors are needed.

## Introduction

Inflammation orchestrates a network of innate and adaptive immune cells. Inflammation starts with the acute phase where innate immune cells encounter a foreign antigen for the first time (1). Prolonged or uncontrolled acute inflammation leads to chronic inflammation which is a more intensive infiltration of various immune cells to the site of inflammation (2). CD4^+^ T cells are key regulators to determine the progress of chronic inflammation by producing cytokines, effector molecules and changing their phenotype. Regulatory aspects of CD4^+^ T cells can vary depending on the environmental cues (1). In this regard, human mesenchymal stem cell (hMSC) therapy might be a promising tool to resolve acute or chronic inflammation in adults and neonates, since these cells have immunomodulatory capacity and regenerative potential. In several clinical studies, hMSC were already proven to be good candidates for cell therapy in inflammatory disorders such as graft versus host disease (GVHD) (3), sepsis (4) inflammatory bowel disease (IBD) (5, 6), and cerebral palsy in neonates (7). However, the effect of hMSC on T cells and its underlying mechanisms are not fully elucidated. There are various *in vitro* data on the mechanism by which these cells exert their immunomodulatory potential; hMSC may act directly by cell-cell contact (8), through paracrine factors (9, 10), or synergic interaction between the two (11). Umbilical cord derived MSCs (UC-MSCs) were shown to inhibit lymphocyte proliferation and IFNγ production against either mitogenic or allogenic stimuli through the paracrine factor, prostaglandin-2 (PGE2) (12). Several other factors secreted by hMSC were identified that can modulate the T cell response, including transforming growth factor beta (TGFβ) (13), and indoleamine 2,3-dioxygenase (IDO) (9), indicating that paracrine factors secreted by hMSC are capable of modulating immune responses. In addition, there is mounting evidence showing that surface interactions between hMSC and immune cells are also necessary for hMSC to exert their immunomodulatory function. More precisely, when bone marrow derived hMSC were co-cultured with soluble anti-CD3 and anti-CD28 monoclonal antibody (mAb) activated T cells, hMSC increased the cell surface expression of adhesion molecules to sustain MSC-T cell adhesion, important for the MSC-mediated immunosuppressive effect. Further, functional blockade of these adhesion molecules diminished the immunosuppressive effect of hMSC (14). Lastly, activated T cells were shown to attach to hMSC and transmigrate underneath them. Depending on the nature of the stimulatory agent, hMSC caused either growth inhibition or lymphocyte proliferation upon attachment (11). Despite these studies, the mechanism by which hMSC modulate T cell responses is not yet fully understood, nor is the effect on CD4^+^ T cells fully elucidated, as most studies focused on CD8^+^ T cells, aiming to interfere with their cytotoxic potential.

In this study, we assessed the immunomodulatory effect of umbilical cord derived MSC (UC-MSC) on T cell phenotype and function, with a focus on activation, proliferation, and maturation of CD4^+^ T cells, aiming to further elucidate the UC-MSC mode of action. Lymphocyte-UC-MSC co-cultures revealed that UC-MSC delayed CD4^+^ T cell proliferation, rather than suppressing it, neither interfering with its activation status nor inducing anergy. Moreover, UC-MSC induced CD4^+^ T cell maturation by skewing the phenotype from effector memory T cells (T_EM_) to central memory T cells (T_CM_). We observed that this activity of UC-MSC required both cell-cell contact and paracrine signaling for a sustainable phenotype which was not dependent on other cell populations. The transient impairment of T cell maturation and differentiation shows promise for clinical feasibility of stem cell therapy.

## Materials and methods

### Umbilical-cord mesenchymal stem cell culturing and harvesting

Umbilical-Cord mesenchymal stem cells (UC-MSC) were provided by Chiesi Farmaceutici S.p.A, Italy. Cells were stored in liquid nitrogen. UC-MSC were received in passage number 4, and not passaged further. For every experiment a new vial was used.

UC-MSC were cultured in complete MSC growth medium provided by Chiesi Farmaceutici S.p.A, Italy which contains 2000U/ml heparin (Leo Pharma B.V., Copenhagen, Denmark) and 5% human platelet lysate (provided by Chiesi Farmaceutici S.p.A, Italy). According to the standard protocol, on the day of UC-MSC seeding, cells were thawed using a 37° C water bath for 4-6 minutes. Cells were centrifuged at 500 x g for 10 minutes at room temperature. Supernatant was removed and 15 mL complete MSC growth medium was added to the cells. Flasks were placed in a 37°C, 5% CO_2_, 95% humidity incubator for 48 h. After 48 h, UC-MSC culture, medium was aspirated, and cells were washed with sterile PBS (Fresenius Kabi, Bad Homburg, Germany). UC-MSC were harvested by using 3 U/ml trypsin/EDTA (Gibco^®^Thermo Fisher Scientific, Waltham, USA). Trypsinization was neutralized by using cold basal MSC culture medium. Cells centrifuged at 300 x g for 10 min at 4°C. Cells were washed after aspirating the medium and counted with trypan blue for further usage.

### UC-MSC inactivation with ß-propiolactone

UC-MSC were seeded in T75 flasks with complete MSC growth medium. 24h later, the cells were stimulated with BPL (Thermo Fisher Scientific) in a 1:200 dilution factor (v/v). BPL-treated cells were incubated at 4° C for 16 h, followed by a 4 h incubation at 37° C to inactivate BPL. After treatment with BPL, cells were washed twice with PBS to remove leftover product. Since BPL fixates the cells, UC-MSC were counted manually using trypan blue staining.

### UC-MSC conditioned medium

UC-MSC were seeded in T75 flasks with complete MSC growth medium, 24h later, complete MSC growth medium was removed and replaced by complete RPMI 1640 (Life technologies), supplemented with 10% human processed serum (HPS) (manufactured in-house), 1 mM pyruvate, 2 mM glutamax, 100 U/ml penicillin, and 100 μg/ml streptomycin (all Thermo Fisher Scientific) for the following 24 h. After a total of 48 h of culture, cellular debris was removed by centrifugation for 15 minutes at 2000 x g, 4°C.

### MTT assay

To evaluate the metabolic activity of BPL-stimulated cells, 10.000 cells were seeded per 96-well flat-bottomed plate (Greiner Bio-One, Frickenhausen, Germany) in 150 µl complete MSC growth medium. Cells were left overnight for attachment and 24 h later, cells were centrifuged at 250 x g for 2.30 minutes, and supernatant was removed. 100 µl fresh culture medium with 10 µl of 12mM 3-(4,5-dimethylthiazol-2-yl)-2,5 ditetrazolium bromide (MTT) (Thermo Fisher) was added to each well. The cells were incubated at 37° C for 4 h. Subsequently, cells were centrifuged again at 250 x g for 2.30 minutes, medium was removed and 50 µl DMSO was added to each well. Plates were incubated at 37° C for 10 minutes. Absorbance was measured at 540 nm at TECAN infinite F50 (TECAN, Switzerland).

### Peripheral blood mononuclear cell isolation and negative selection of CD4+ T cells

Blood samples for peripheral blood mononuclear cell (PBMC) isolation were collected in 10 ml EDTA tubes (BD, Labware, NJ). Blood was obtained from healthy adult individuals and prior to sampling blood, donors signed a written informed consent for scientific use according to Dutch law. PBMC were isolated using density gradient centrifugation (Lymphoprep, Nycomed Pharma AS, Oslo, Norway). CD4^+^ T cells were isolated by negative selection using Miltenyi Biotech CD4^+^ T cell Isolation Kit (Miltenyi Biotec GmbH, Bergisch Gladbach, Germany), according to manufacturer’s protocol. The purity of the sorted CD4^+^ T cells was typically above 95%. Viability of PBMC and CD4 was above 80% at the beginning of the co-cultures. Isolated cells were cultured in complete RPMI 1640 medium.

### CD4+ T cell stimulation and UC-MSC co-culture

In our preliminary studies, two UC-MSC : PBMC cell ratios were tested, 1:2.5 and 1:5. CD4^+^/CD8^+^ ratio, CD4^+^Ki67^+^ and CD8^+^Ki67^+^ percentages were tested, whereby the results using the 1:5 UC-MSC : PBMC ratio were more outspoken (data not shown). Therefore, the following experiments were performed with the 1:5 ratio (UC-MSC: PBMC or UC-MSC: CD4^+^).

100.000 CD4^+^ T cells were seeded in 96 well U-bottom plates (Greiner Bio-One) with complete RPMI medium. CD4^+^ T cells were activated with αCD3/CD28 beads (Dynabeads™ Human T-Activator, Gibco®Thermo Fisher Scientific) in 1:5 (bead-to-cell) ratio. After 48 h of culture 20.000 UC-MSC were seeded in 96-well U-bottom plates with complete MSC growth medium. Both CD4^+^ T cells and UC-MSC were kept in culture for 24 h to allow for activation and attachment, respectively. The following day, growth medium of UC-MSC was removed, CD4^+^T cells were resuspended in complete RPMI medium and transferred onto seeded UC-MSC. Cells were cultured together for up to 5 days, and flow cytometry measurement was acquired every other day.

### Transwell system

250.000 PBMCs were seeded into 24 well plates (Greiner Bio-One) and activated with αCD3/CD28 beads (Dynabeads™ Human T-Activator, Gibco®Thermo Fisher Scientific) in 1:5 (bead-to-cell) ratio. In addition, 48h after pre-culturing, 50.000 UC-MSC were seeded in transwell inserts (ThinCerts™ Greiner Bio-One) with complete MSC growth medium and allowed to adhere overnight. 24h later, complete MSC growth medium was removed from the transwell inserts, and medium replaced by complete RPMI medium. Inserts were placed onto wells containing αCD3/CD28 activated PBMC. Cells were cultured for up to 5 days.

### Flow cytometry and cell sorting

Samples were transferred to V-bottom plates (Greiner Bio-One) and washed once with flow cytometry buffer (FACS Buffer, containing 0.2% bovine serum albumin, BSA (Sigma-Aldrich, St. louis, USA), in PBS (Fresenius Kabi)). Cells were first stained with viability dye for 30 minutes at 4°C, then washed once with FACS Buffer. For surface staining, samples were stained with monoclonal antibodies of interest for 20 minutes at room temperature in the dark. Cells were washed twice with FACS Buffer. For intracellular staining, cells were permeabilized and fixed according to manufacturer’s instructions (eBioscience, San Diego, USA). Fixed and permeabilized cells were washed once with permeabilization buffer. Samples were incubated with conjugated antibodies for 30 minutes at 4°C in the dark. As a last step, cells were washed twice with permeabilization buffer, and transferred to FACS tubes for acquisition using the Navios™ flow cytometry (Beckman-Coulter, Fullerton, CA, USA).

For intracellular cytokine expression, cells were stimulated with phorbol-12-myristate-13-acetate (PMA), ionomycin and brefeldin A (respectively, 12.5 ng/ml, 500 ng/ml, and 5 µg/ml; all from Sigma-Aldrich, St. Louis, USA) for 4 h, at 37° C in a humidified 5% CO_2_ incubator.

**Supplementary Table 1** summarizes the antibodies used for flow cytometry and sorting. The average viability at the end of co-culture of unstimulated PBMC was always >60%; stimulated PBMC had a viability of >75%; unstimulated CD4^+^ T cells >70% and stimulated CD4^+^ T cells >80%.

### T cell proliferation assay

CD4^+^ T cells were sorted on a FACSAria III (BD Bioscience, San Jose, CA, USA) at room temperature into polypropylene round bottom Falcon tubes. Sorted cells were rested for 24h. The following day, samples (1-1.5 x 10^6^ cells) were labelled with 0.1 µM carboxyfluorescein succinimidyl ester (CFSE; Gibco®Thermo Fisher Scientific). After labelling, 50.000 CD4^+^T cells were seeded per condition. Samples were stimulated with either 100 U/mL IL2 or 1:5 (bead-to-cell ratio) αCD3/CD28 beads (Dynabeads™ Human T-Activator, Gibco®Thermo Fisher Scientific). Phenotypical changes were acquired after 24h and 48h with flow cytometry.

### Cytokine analysis

Cytokine levels of the supernatant of cell culture as determined using MILLIPLEX Human Cytokine/Chemokine Magnetic Bead Panel (Merck Darmstadt, Germany). The following analytes were measured: IL-1β, IL-2, IL-4, IL-6, IL-10, IL-12p70, IL-17A, TNFα, TNFβ, CCL4, CCL11 (Eotaxin), CX3CL1, MCP3, IFNγ, and CXCL10 (IP-10). The assay was performed according to manufacturer’s protocol. Data acquisition was performed on the FlexMap-3D System (Luminex) using xPONENT Software (Luminex).

For TGFβ, cell culture supernatants were analyzed using an ELISA Kit (R&D System, Minnesota, USA), performed according to manufacturer’s recommendations.

### IL-6 and pan-TGFβ neutralization

Neutralizing IL-6 (R&D System) or Pan-TGFβ (R&D System) monoclonal antibodies were added to 24h αCD3/CD28 bead activated CD4^+^ T cells, 30 minutes prior to the start of the co-culture with UC-MSC. Based on a pilot dose response experiment, 1 ug/mL and 10 ug/mL neutralizing IL-6 and Pan-TGFβ were used, respectively in subsequent experiments. Neutralizing antibodies were administered each day of the culture. Phenotypic analysis was performed after 3 days of co-culturing in the presence or absence of neutralizing antibodies.

### Analysis

Flow Cytometry data were analyzed using Kaluza Software (Beckman Coulter, analysis version 2.1). Graphics and statistical tests were performed in R version 3.6.2, version 1.6.0. Differences between the levels of outcome were explored using paired-t-test with mean average. The level of statistical significance was set at *p<0.05, **p<0.01, ***p<0.001, ****p<0.0001.

## Results

### Differential effects of UC-MSC on CD4+ T cells in PBMC versus sorted CD4 T cell co-cultures

Previously, it was shown that hMSC were able to suppress proliferation of T cells in a mixed lymphocyte reaction (13) or incubation with TCR-specific activators (αCD3/CD28 beads) (15). While some studies suggest elevated CD25 expression of T cells in hMSC cocultures (16), others show a reduction (17). Considering these inconclusive data, we examined the UC-MSC regulated proliferation and activation of T cells in more detail.

**Figure 1:**
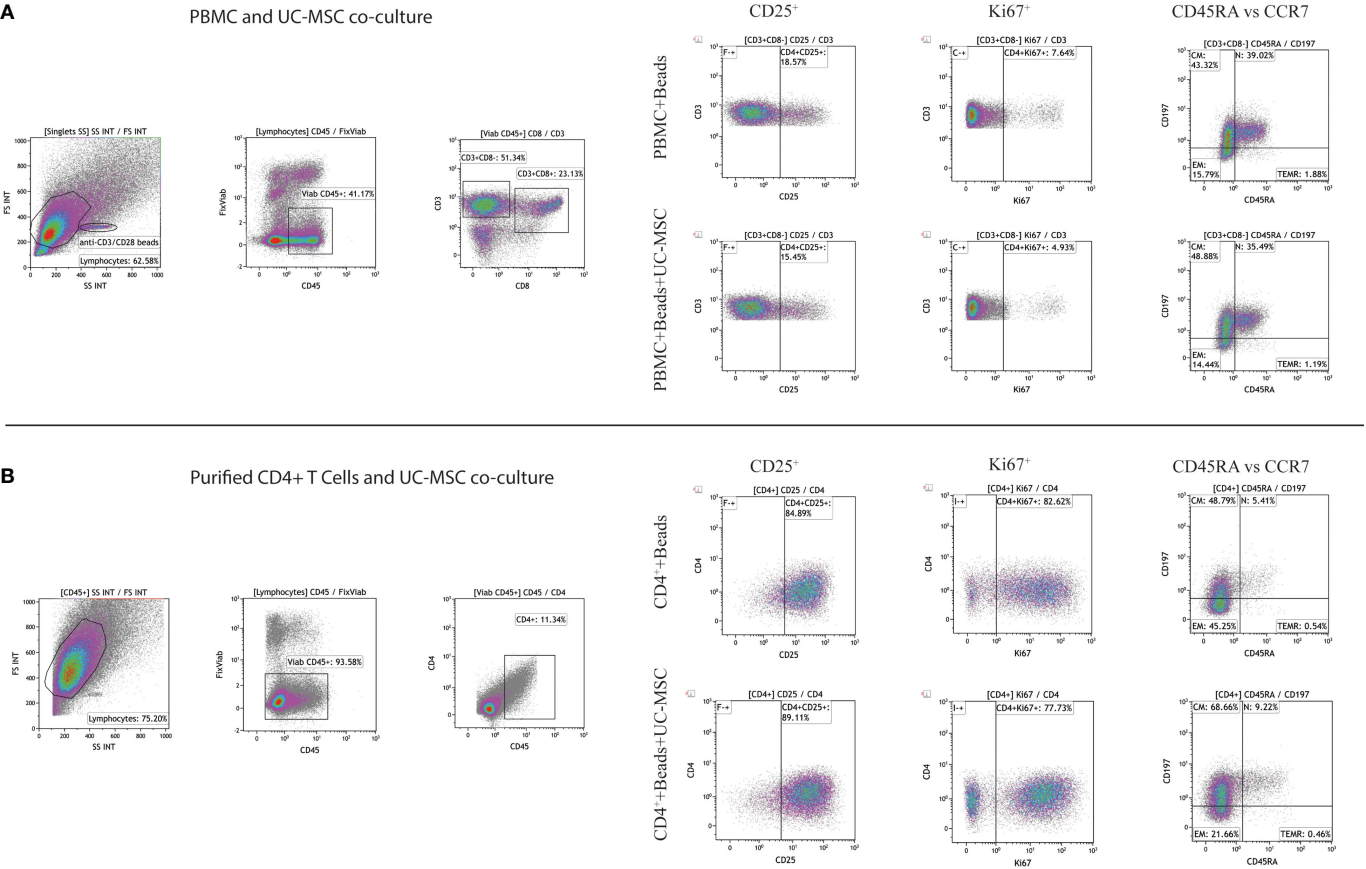
Gating strategy of CD4^+^ T cells. Representative gating strategy for assessment of CD25, Ki67, and memory T cell subsets in PBMC and UC-MSC cocultures **(A)** and CD4^+^ T cells and UC-MSC co-cultures were shown **(B)**. Corresponding figures are from day 1 of co-cultures. Gate positions were selected by using unstimulated cells.

**Figure 2:**
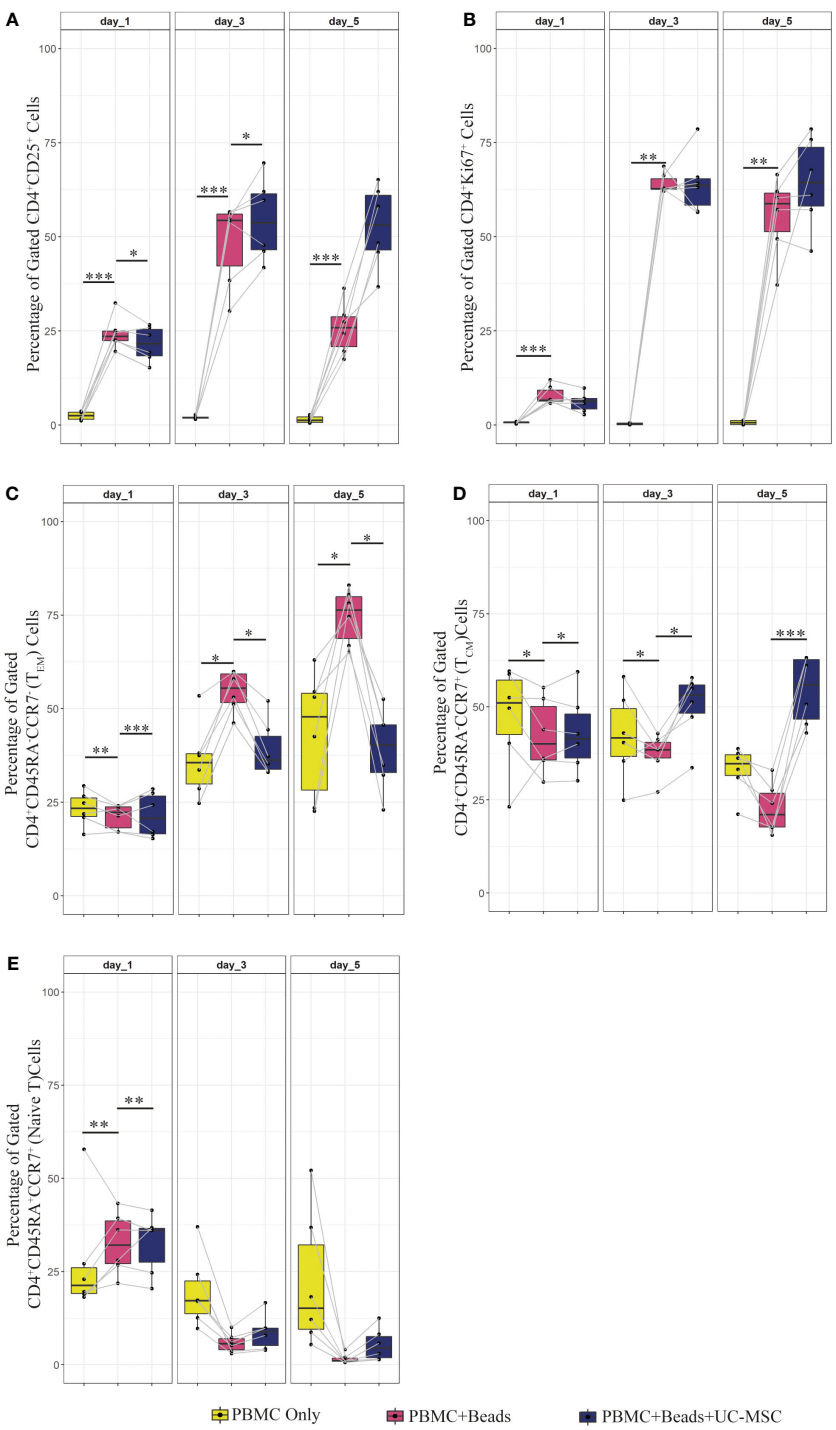
Proliferation, activation and memory formation of T cells in PBMC co-culture with UC-MSC. PBMC were stimulated with αCD3/CD28 beads (PBMC+Beads) in a 1:5 ratio (bead:cell). Unstimulated cells (PBMC Only) were used as negative control. 24 hours after stimulation, UC-MSC were added to PBMC+Beads cultures (PBMC+Beads+UC-MSC) in a 1:5 (UC-MSC : PBMC) ratio. Changes in cell phenotypes were acquired every other day for in total 5 days. Representative flow cytometry plots and gating strategy of the PBMC co-cultures can be found in **Figure 1A**. Frequencies of different T cell subsets are shown: CD4^+^CD25^+^ **(A)**, CD4^+^Ki67^+^ **(B)**, CD4^+^CD45RA^-^CCR7^-^ (effector memory T cells; T_EM_) **(C)**, CD4^+^CD45RA^-^CCR7^+^ (central memory T cells; T_CM_) **(D)**, and CD4^+^CD45RA^+^CCR^+^ (Naive) T cells **(E)**. 7 biological replicates are used in the graphs and the paired-t-test was used to assess differences between each group; *p<0.05, **p<0.01, ***p<0.001.

PBMC (PBMC Only) were pre-stimulated with αCD3/CD28 mAb (αCD3/CD28 beads) for 24 hours (PBMC+Beads), after which UC-MSC were added (PBMC+Beads+UC-MSC). Phenotypic changes were measured every other day for 5 days (see **Supplementary Figure 1A** for the experimental setup). **Figure 1A** shows the gating strategy for PBMC and UC-MSC co-cultures. Lower percentages of CD4^+^CD25^+^ T cells were found on day 1 and day 3. This effect was diminished on day 5 (**Figure 2A**). UC-MSC were not able to inhibit αCD3/CD28 induced proliferation, as measured by CD4^+^Ki67^+^ expression (**Figure 2B**). Furthermore, T cell maturation was investigated in these same co-cultures. We observed a lower percentage of effector memory T cells (T_EM_, CD45RA^-^CCR7^-^) from day 1 onwards (**Figure 2C**), while an increase in central memory T cells (T_CM_, CD45RA^-^CCR7^+^) was observed in the PBMC+Beads+UC-MSC condition (**Figure 2D**). The percentage of naïve T cells (**Figure 2E**) was also higher in the presence of UC-MSC, albeit at lower levels than T_EM_ and T_CM_.

**Figure 3:**
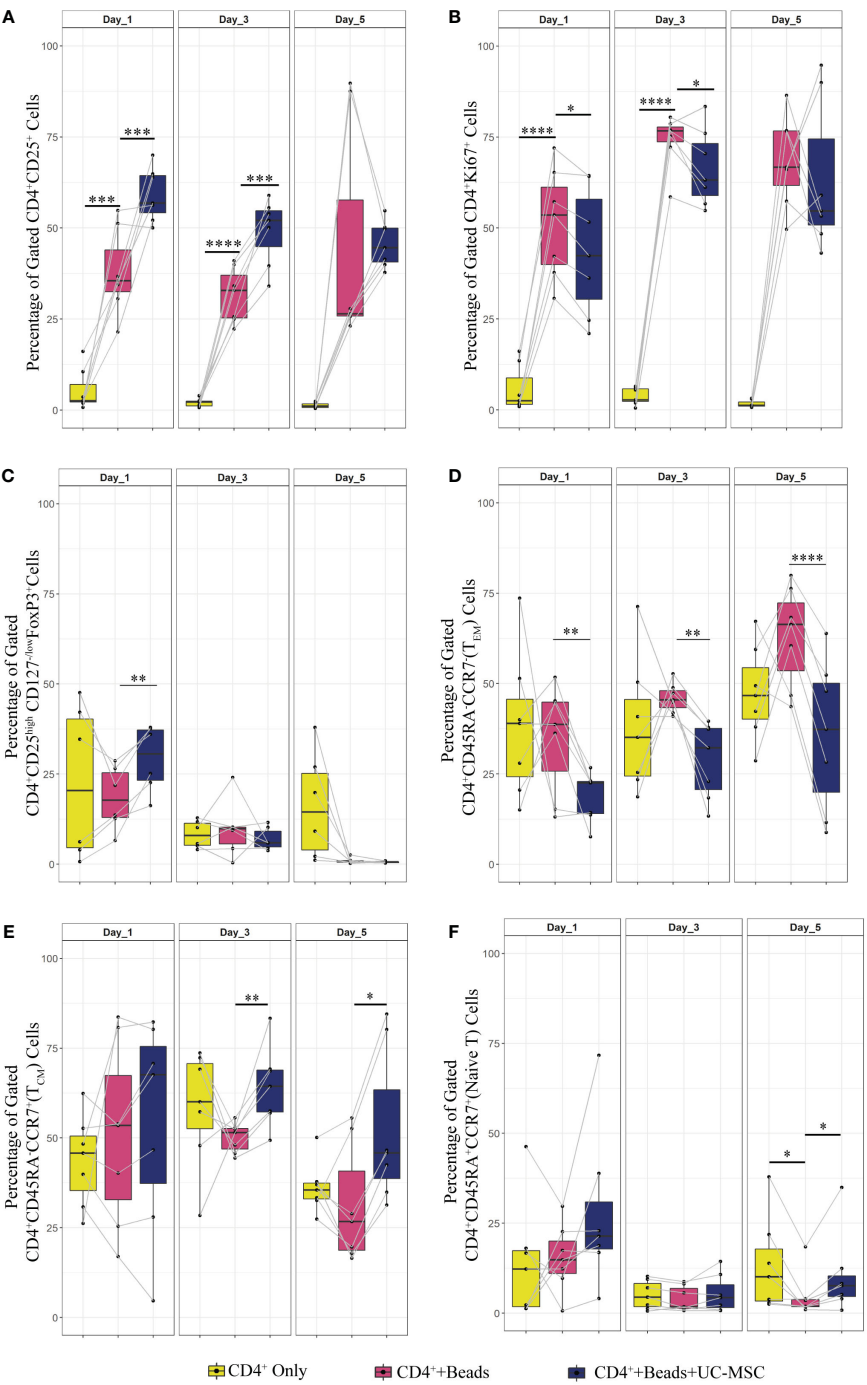
Effect of UC-MSC on activation, proliferation and maturation of negatively selected CD4+ T cells. CD4+T cells were negatively selected from the PBMC pool. They were either stimulated with αCD3/CD28 beads (CD4^+^+Beads) or kept unstimulated (CD4+ Only). 24 hours after stimulation, UC-MSC were added to the culture system (CD4^+^+Beads+UC-MSC). Phenotypical changes were acquired every other day for 5 days. Representative flow cytometry plots and gating strategy of the CD4^+^ co-cultures can be found in **Figure 1B**. Percentages of CD4^+^CD25^+^ **(A)**, CD4^+^Ki67^+^ **(B)**, CD4^+^CD25^high^CD127^-/low^ FOXP3^+^ **(C)**, CD4^+^CD45RA^-^CCR7^-^ (T_EM_) **(D)**, CD4^+^CD45RA^-^CCR7^+^ (T_CM_) **(E)**, and CD4^+^CD45RA^+^CCR^+^ (Naive) T cells **(F)** are shown. 7 biological replicates are included and the paired-t-test was used to assess differences between each group; *p<0.05, **p<0.01, ***p<0.001, ****p<0.0001.

To check whether the observed effects were exclusively dependent on the interaction of UC-MSC with T cells and not through additional by-stander cells in the PBMC mixture, we purified CD4^+^ T cells (CD4^+^ Only) by negative selection and then set up co-cultures with UC-MSC. Thus, 24-hour pre-activated purified CD4^+^ T (CD4^+^+Beads) cells were co-cultured with UC-MSC (CD4^+^+Beads+UC-MSC) for 5 days. Flow cytometric measurements were acquired every other day (**Supplementary Figure 1A**). **Figure 1B** shows the gating strategy for CD4^+^T and UC-MSC co-cultures. Again, we observed a further increase in the percentage of CD4^+^CD25^+^ activated T cells in the presence of UC-MSC (**Figure 3A**). Proliferation of the CD4^+^ cells was reduced by the addition of UC-MSC, mostly in the early stages of culture, but not anymore on day 5, which would point to a delay in proliferation rather than complete suppression (**Figure 3B**). Lastly, addition of UC-MSC led to a higher percentage of CD4^+^CD25^high^CD127^low/neg^FOXP3^+^ activated T cells (**Figure 3C**), but only on day 1; on the following days, this population was no longer present, pointing towards a short-lived activation rather than the induction of a putative regulatory cell type.

These results suggest that expression of the activation marker, CD25, was impaired in PBMC co-cultures for a short-term, and that the effect was not observed when CD4^+^ purified cells were used. Proliferation was delayed only in the co-cultures set up with CD4^+^ purified cells.

Regarding the effect of UC-MSC on the maturation state of T cells, the effect was most pronounced using purified CD4^+^ T cells in the co-cultures. From day 1 onwards, the CD4^+^ T_EM_ fraction was reduced in the presence of UC-MSC (**Figure 3D**). In contrast, on day 3 and 5, the CD4^+^ T_CM_ fraction increased in UC-MSC co-cultures (**Figure 3E**). The naïve T cell population showed an increase on day 5, yet the increase was not as high as seen for T_CM_ (**Figure 3F**). There was no significant change in terminally differentiated effector memory cells (data not shown). Thus, in both PBMC and purified CD4^+^ T cell co-culture settings, T_EM_ started to decrease from day 1 onwards, whereas an increase in T_CM_ was most apparent from day 3 onwards.

### UC-MSC-mediated immunomodulation occurs through paracrine and direct cell-cell contact

Having established that UC-MSC interfere with T cell memory formation, we further investigated a possible underlying mechanism. Previously published studies provided contradicting evidence regarding the involvement of direct cell contact versus paracrine factors in the immunomodulatory function of MSC on T cells (10, 13, 14, 18–23). Here, the effect of direct cell-cell contact (DCC) was examined by co-culturing αCD3/CD28 beads pre-stimulated PBMC with UC-MSC over a 5-day period. The effect of UC-MSC derived paracrine factors was investigated by either using a transwell system (TW), with 400 nm pores or the addition of culture medium derived from UC-MSC (to be able to capture factors greater than 400 nm; conditioned medium, CM). 250.000 αCD3/CD28 bead stimulated PBMC were co-cultured with either UC-MSC (DCC or TW) or UC-MSC derived conditioned medium in final concentrations of 25% and 50% (CM50% and CM25%, respectively). Phenotypic changes were measured every other day over a period of 5 days (**Supplementary Figure 1A**). TW and CM conditions were then compared to the DCC condition.

**Figure 4:**
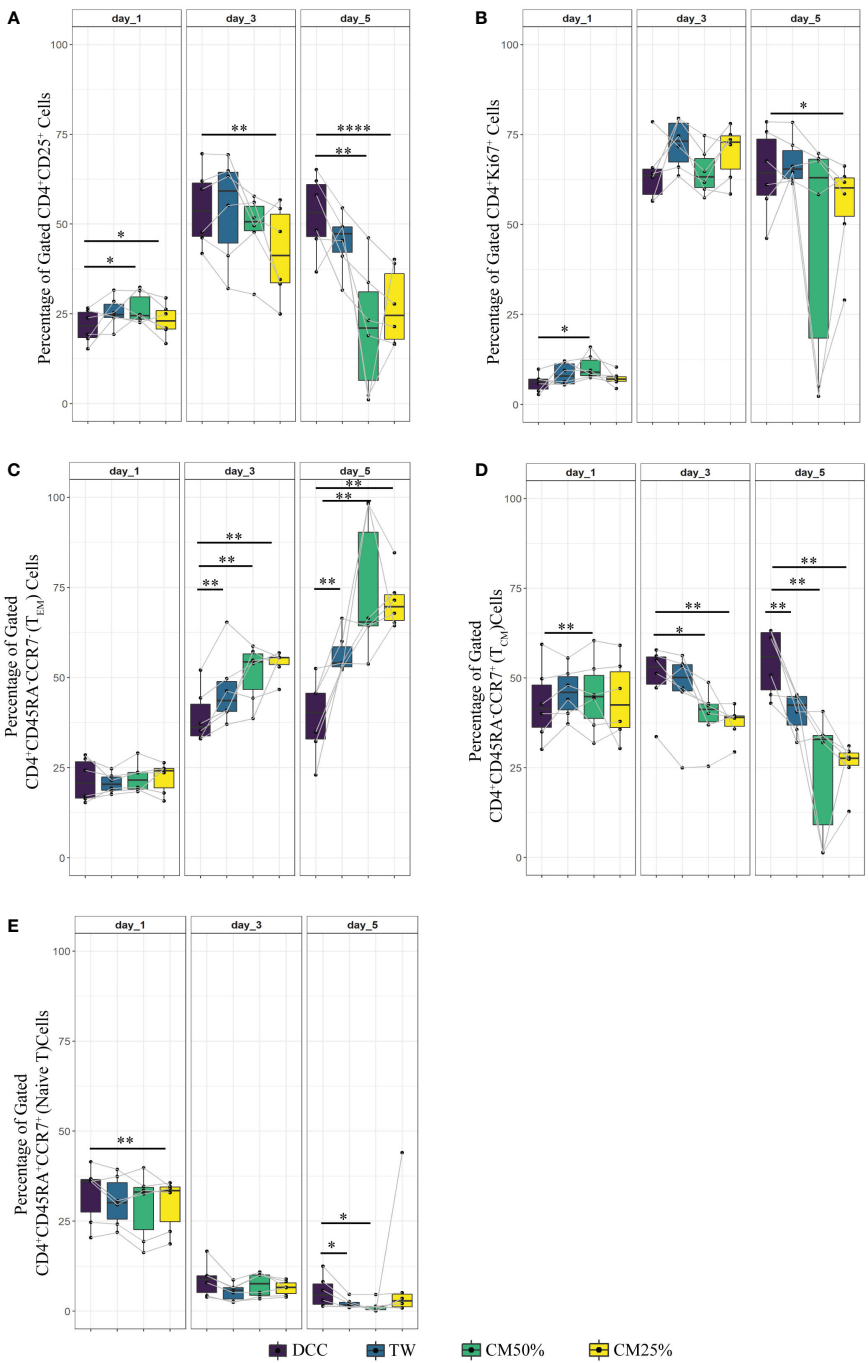
Comparison of the UC-MSC dependent mechanisms involved in T cell regulation. 24 hours after activating PBMC with αCD3/CD28 beads, activated PBMC were exposed to UC-MSC in either direct cell-cell contact (DCC), transwell (TW) or with UC-MSC derived condition medium (CM) with a final volume of 25% or 50% (CM25% or CM50%, respectively). Percentages of different T cell subsets at day1, 3 and 5 of co-culture are shown: CD4^+^CD25^+^ **(A)**, CD4^+^Ki67^+^ **(B)**, CD4^+^CD45RA^-^CCR7^-^ (T_EM_) **(C)**, CD4^+^CD45RA^-^CCR7^+^ (T_CM_) **(D)**, and CD4^+^CD45RA^+^CCR^+^ (Naive) **(E)**. 7 biological replicates are included and the paired-t-test was used to assess differences between each group; *p<0.05, **p<0.01, ****p<0.0001.

Regarding the expression of the activation marker CD25, the TW, CM50% or CM25% conditions revealed a trend towards higher levels on day 1 compared to DCC, but this effect did not persist. There were more CD25^+^ T cells in DCC compared to other conditions (**Figure 4A**). Although **Figure 2** revealed a trend towards reduction in proliferation in the DCC condition (PBMC+Beads+UC-MSC) on day 1, **Figure 4B** shows that none of the other conditions led to significant suppression of proliferation (Ki67 expression) in αCD3/CD28 beads activated CD4^+^ T cells. As compared to DCC, higher percentages of proliferating (Ki67^+^) cells were found in TW and the CM conditions.

Subsequently, the T cell memory formation was investigated. **Figure 2** showed that the direct presence of UC-MSC efficiently inhibits T_EM_ formation. TW or the addition of CM50% or CM25% resulted in a less reduction of T_EM_, with significantly higher percentages of T_EM_ compared to DCC (**Figure 4C**). In all conditions a similar percentage of T_CM_ was present on day 1 of culture. However, over time, in none of the conditions T_CM_ level was maintained to the degree found in DCC (**Figure 4D**). The frequency of the naïve T cell population was also higher in DCC, compared to the other conditions (**Figure 4E**).

These results suggest that both direct UC-MSC interaction with T cells and UC-MSC-derived factors modulated T cell activation as measured by CD25 expression, while only direct contact delayed proliferation. UC-MSC clearly affected memory T cell formation, whereby direct cell contact efficiently suppressed T_EM_ and increased T_CM_ percentages, and paracrine factors failed to significantly affect T_EM_, and T_CM_ formation.

### Surface interaction is not a key modulator for phenotypical changes in CD4+ T cells

Various methods have been proposed to investigate the contribution of surface interactions in cell function, such as heating (24), γ-irradiation (25), and chemical agents (e.g. formaldehyde) to block the secretion and proliferative capabilities of the cells (26). Here, we used beta-propiolactone (BPL), effectively inactivating cells by crosslinking DNA with proteins, thereby blocking secretion from UC-MSC, including cytokine secretion, but still allowing cell-cell interaction (27). BPL inactivation causes relatively low damage to surface antigens as compared to other methods (26). UC-MSC were inactivated with BPL (BPL-UC-MSC) for 24h (**Supplementary Figure 1A**). To confirm that UC-MSC were metabolically inactive a MTT assay was conducted (**Supplementary Figure 1B**).

**Figure 5:**
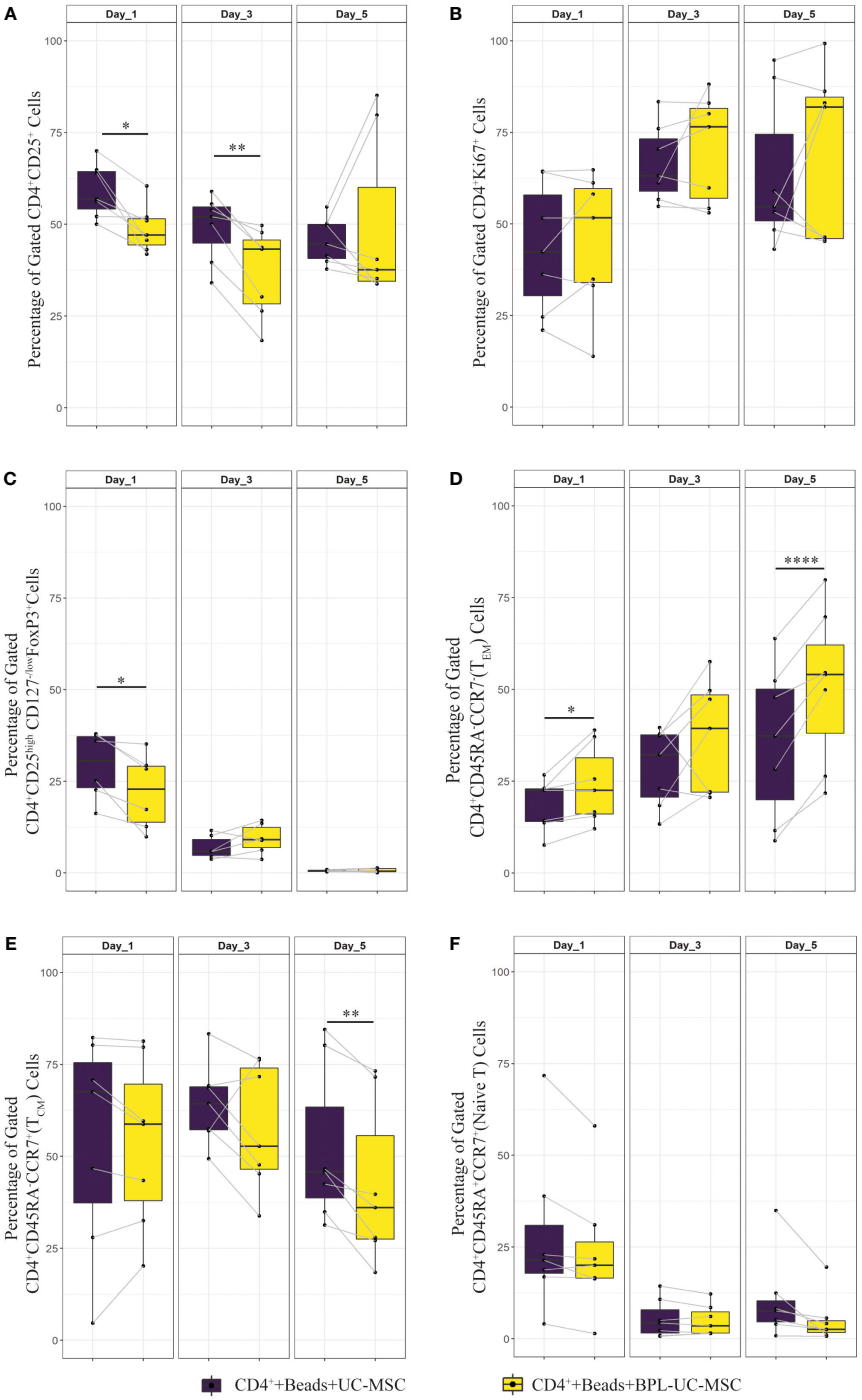
Immunomodulatory effect of surface interaction between UC-MSC and CD4^+^ T cell. Negatively selected CD4^+^ T cells were stimulated 24 hours with αCD3/CD28 beads. CD4^+^ T cells were co-cultured with either UC-MSC (UC-MSC) or BPL treated UC-MSC (BPL-UC-MSC). 24 hours BPL treatment of UC-MSC was used to block paracrine factor secretion to evaluate the impact of surface proteins on immunomodulation. The effect of this blockade on T cell regulation was validated by measuring phenotypical changes of CD4^+^ T cells. Frequencies of different T cell subset were determined at day 1, 3 and 5 of co-culture: CD4^+^CD25^+^ **(A)**, CD4^+^Ki67^+^ **(B)**, CD4^+^CD25^high^CD127^-/low^ FOXP3^+^ **(C)**, CD4^+^CD45RA^-^CCR7^-^ (T_EM_) **(D)**, CD4^+^CD45RA^-^CCR7^+^ (T_CM_) **(E)**, and CD4^+^CD45RA^+^CCR^+^ (Naive) T cells **(F)**. In all graphs, 7 biological replicates are included and the paired-t-test was used to assess differences between each group; *p<0.05, **p<0.01, ****p<0.0001.

**Figure 5A** shows that removing UC-MSC specific paracrine factors from the co-culture (CD4^+^+Beads+BPL-UC-MSC), prevents the increase in the percentage of CD25^+^ T cells compared to the CD4^+^+Beads+UC-MSC condition. Proliferation rates (% of Ki67^+^ cells) were similar for both conditions (**Figure 5B**). In the early activated CD4^+^ T cell population, BPL-treated MSC led to a significantly lower percentage of CD4^+^CD25^high^CD127^low/neg^FOXP3^+^ cells compared to untreated UC-MSC (**Figure 5C**). Notably, BPL-UC-MSCs were not able to reduce the T_EM_ and induce the T_CM_ populations to levels observed for untreated UC-MSC (**Figures 5D, E**), with only significant differences on day 5 of co-culture (**Supplementary Figures 2A, B**). There was no significant change in the naive T cell population (**Figure 5F**).

Overall, these findings suggest that surface marker interaction between UC-MSC and T cells alone led to lower level of CD25^+^ T cells. Moreover, there were less CD25^high^ cells with a possibly regulatory phenotype. Also, surface interaction alone (BPL-treated UC-MSC) was not as effective as the combined effect of surface interaction with paracrine factors (UC-MSC) on the induction of memory formation in CD4^+^ T cells.

### IL-6 and TGFβ are the most abundantly expressed cytokines in the co-cultures

Previously, it was shown that hMSCs were able to reduce the pro-inflammatory mediators such as IL-1β, IFNγ, IL-6, TNFα and VEGFα; and increase TGFβ, and prostaglandin E_2_ (PGE_2_) (23, 28, 29) levels. Abundance of these paracrine factors varies under different experimental and disease conditions [reviewed in (30)]. In order to assess the impact of UC-MSCs in our co-culture system, selected cytokines or chemokines were measured by either ELISA or Luminex.

**Figure 6:**
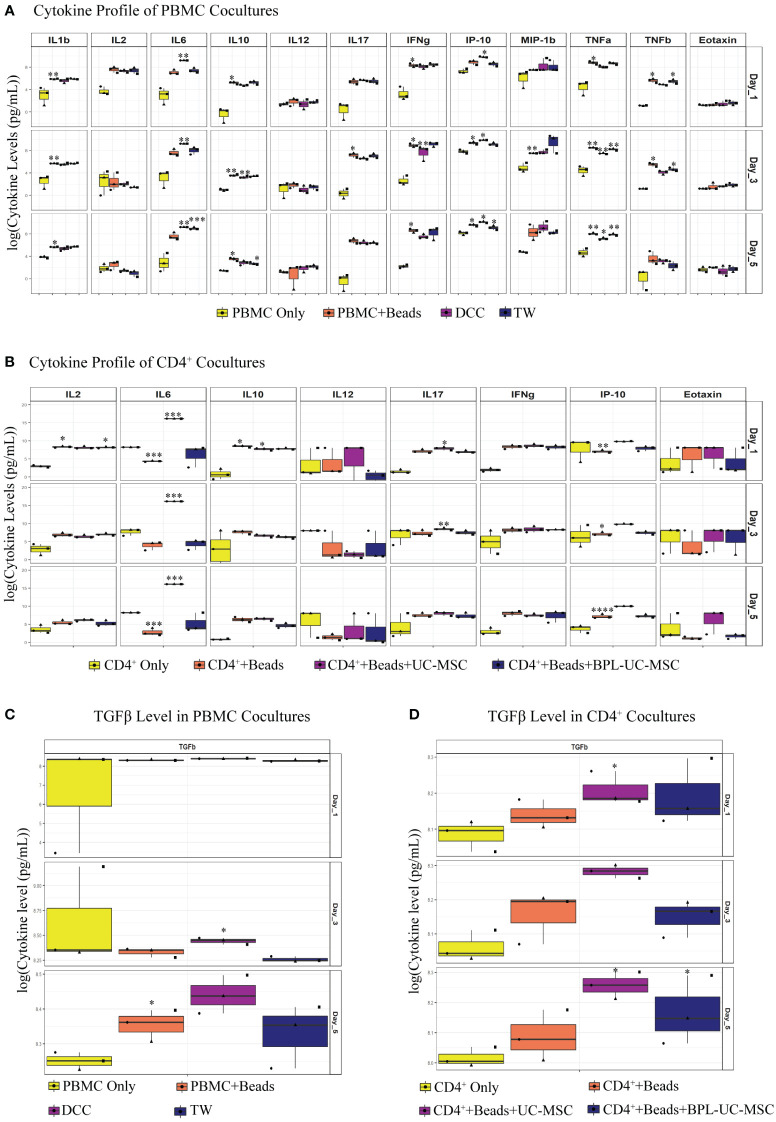
Soluble factors were analyzed in UC-MSC co-cultures with either PBMC or purified CD4^+^ T cell. Soluble mediators were analyzed via Luminex immunoassay: IL-1β, IL-2, IL-6, IL-10, IL-12, IL-17, IFNγ, IFNγ-induced protein 10 (IP-10), macrophage inflammatory protein 1β (MIP-1β), TNFα, TNFβ, and Eotaxin. TGFβ levels were measured by ELISA. Differences between groups were expressed in (log10) fold change on the y-axis. Luminex assay was performed on co-culture supernatant of both PBMC **(A)** and purified CD4^+^ T **(B)** experiments. Similarly, TGFβ levels were measured for both PBMC **(C)** and pure CD4^+^ T **(D)** cell culture supernatant. In the conditions where the concentration was above or below the detection limit, maximum or minimum detectable concentration were used, respectively. Analytes that did not have proper standard curves were excluded from the analysis. 3 biological replicates are shown in all graphs. *p<0.05, **p<0.01, ***p<0.001, ****p<0.0001 (data are compared using the paired-t-test between each group).

In the DCC (PBMC+Beads+UC-MSC) condition (**Figure 6A**), IFNγ expression was significantly reduced on day3. Although not significant, the concentration of IFNγ at day 5 was lower in the DCC conditions compared to beads stimulated PBMC. Although IL-2 levels did not show any significant reduction, there was a trend towards a reduction in IL-2 concentration in the DCC condition at day 5. Further, IL-6 levels in the DCC condition were significantly higher over the whole period of co-culture. We found that the TNFα concentration was significantly reduced from day 1 onwards in the DCC condition. The presence of chemokine factors in culture medium was evaluated by measuring IFNγ-induced protein 10 (IP-10 or CXCL-10) and Eotaxin levels. IP-10 was elevated in the DCC condition from day 1 onwards, whereas no changes were observed in Eotaxin levels. Without direct cell contact, in TW conditions, there was a trend towards a reduction in IL-2 concentration at day 5, and IL-6 was significantly elevated only at day 5. TNFβ was only reduced in TW on day 1 and day 3. Lastly, chemokine factor, IP-10 was significantly elevated at day 5. The dynamics and profiles of cytokine secretion were different between DCC and TW.

Similar to PBMC co-cultures, IL-6 was elevated in CD4^+^+Beads+UC-MSC. IL-17 was significantly higher in the CD4^+^+Beads+UC-MSC condition on day 1 and 3. Regarding IP-10, there was no significant increase in UC-MSC conditions compared to αCD3/CD28 beads activated cells alone, yet the concentration of IP-10 was elevated on all days in CD4^+^+Beads+UC-MSC. Although, we clearly observed reductions in intracellular IFNγ and IL-2 expression (**Supplementary Figures 2C, D**) in T_EM_ from the UC-MSC co-cultures, no significant changes were measured in the culture supernatant, this also held true for IL-12 and Eotaxin. Interestingly, there was no concrete change in IL-6 levels in the BPL-treated UC-MSC and αCD3/CD28 beads activated conditions (**Figure 6B**). This suggests that - as expected- IL-6 was mainly produced by UC-MSCs.

In PBMC co-cultures, on day 3, there was increased TGFβ expression in DCC, but not in TW. On day 5, there was a trend towards elevated TGFβ in DCC (**Figure 6C**). In addition to this, in CD4^+^ co-cultures, TGFβ was significantly induced on day 1 and day 5 in CD4^+^+Beads+UC-MSC. Although, there was no significance on day 3, there was a higher TGFβ concentration in the CD4^+^+Beads+UC-MSC condition (**Figure 6D**).

**Figure 7:**
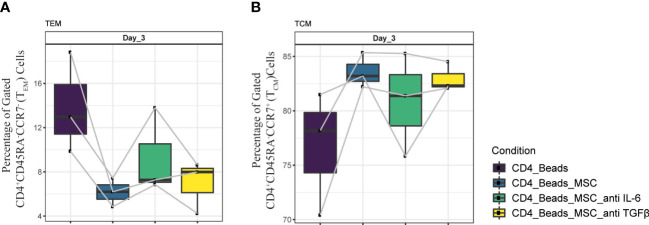
Neutralization of IL-6 and TGFβ in UC-MSC co-cultures with CD4^+^ T cell. Neutralizing IL-6 and TGFβ antibodies were added to 24h pre-activated CD4^+^ T cell cultures, 30 minutes prior to co-culture initiation. The effect of this neutralization on CD4^+^ T cells was validated by acquiring changes in **(A)** CD4^+^CD45RA^-^CCR7^-^ (T_EM_) and **(B)** CD4^+^CD45RA^-^CCR7^+^ (T_CM_) populations. 3 biological replicates are shown and the paired-t-test was used to assess differences between each group.

Overall, a difference in cytokine pattern was observed in PBMC and purified CD4^+^ T cell cultures, with more pronounced differences in the PBMC co-cultures. The most striking changes were found for IL-6, IP-10, TGFβ and TNFα. Since, IL-6 and TGFβ were shown to be explicitly secreted by UC-MSC, we next examined whether these two factors played a role in the phenotypic changes observed in the UC-MSC CD4^+^ T cell co-cultures. Therefore, IL-6 and TGFβ were captured in the co-cultures by using neutralizing antibodies. Even though there was no statistically significant difference, neutralization of IL-6 showed a trend toward higher T_EM_ percentages (**Figure 7A**) and conversely lower levels of T_CM_ (**Figure 7B**). TGFβ showed a similar pattern as IL-6, but to a lower intensity. These results are suggestive that IL-6 and TGFβ may have a role in the immunomodulating effect of UC-MSC on T cell memory formation, but that other paracrine factors and/or surface interactions are also required.

### UC-MSC primed T_CM_ population is responsive upon restimulation

**Figure 8:**
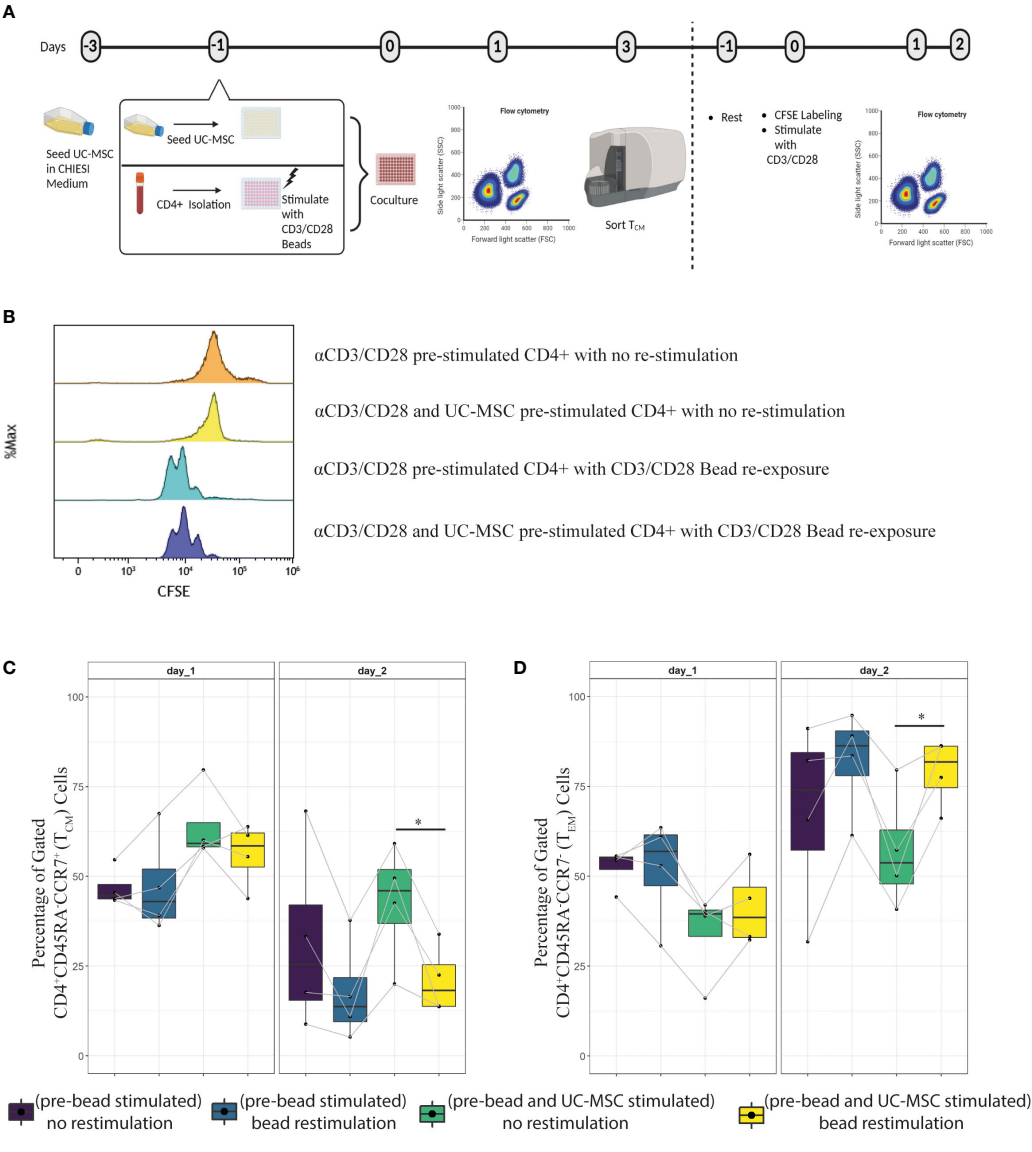
Transient effect of UC-MSC on CD4^+^T cells. The T_CM_ population obtained from day 3 culture of CD4^+^+Beads and CD4^+^+Beads+UC-MSC were FACS sorted for viable CD45^+^CD4^+^CD62L^+^ cells. Sorted cells were labeled with fluorescence dye CFSE, and stimulated with or without αCD3/CD28 beads in the absence of UC-MSC **(A)**. Cell division was measured by flow cytometry 48-hour after labeling and with or without αCD3/CD28 beads re-stimulation **(B)**. The phenotypical switch from central memory **(C)** to effector memory **(D)** was acquired by flow cytometry measurement. 4 biological replicates are shown and the paired-t-test was used to assess differences between each group; *p<0.05.

Having established that the T_EM_ population was reduced, T_CM_ numbers increased, and cytokine production was partially impaired upon co-culture with UC-MSC, it remained to be determined to what extent the UC-MSC induced central memory cells were responsive upon re-stimulation. To this end, after 3 days culture of αCD3/CD28 beads activated CD4^+^ T cells with or without UC-MSC, viable CD45^+^CD4^+^CD62L^+^ (T_CM_) cells were sorted and rested for 1 day. After 24 hours, cells were stained with CFSE for subsequent measurement of proliferation and re-stimulated with αCD3/CD28 beads. 24 and 48 hours after stimulation, CFSE levels, as well as CD45RA and CCR7 expression were measured (**Figure 8A**).

The results showed that the sorted T_CM_ cells were able to proliferate upon restimulation with αCD3/CD28 beads after 48 hours (**Figure 8B**). Furthermore, there was no difference in T_CM_ proliferation between UC-MSC primed and not primed.

When examining the differentiation potential of these cells, we observed that 24 hours later, there was no significant change. However, after 48 hours, there were less T_CM_ and more T_EM_ in pre-UC-MSC-beads exposed and re-stimulated cells, compared to non-re-stimulated cells (**Figures 8C, D**, respectively). There was no difference in T_EM_ and T_CM_ after 48 hours, upon re-stimulation of pre-UC-MSC-beads exposed and pre-beads exposed cells. These findings highlight a phenotypical skewing from T_EM_ to T_CM_ when UC-MSC are present in the co-culture and this effect is reversible after re-stimulation in the absence of UC-MSC.

## Discussion

Chronic inflammation develops as part of a sequence of events following acute inflammation (31). A derailment in the activation and differentiation of T cells, which are key regulators of adaptive immune responses, may contribute to the pathology of chronic disease. Stem cell treatment, previously shown to affect T cell function (32, 33), can therefore be an appealing therapeutic approach to ameliorate the consequences of systemic inflammation. Here, we examined the long-term *in vitro* effect of human umbilical cord derived stem cells (UC-MSC) on activated CD4^+^ T cells in more detail.

We observed that UC-MSC delayed proliferation when co-cultured with purified pre-activated CD4^+^ T cells. The cells did allow for activation and phenotypic shifts, promoting central memory formation and suppressing effector memory function. Also, cytokine production profiles changed upon interaction with UC-MSC. The magnitude of this effect was more pronounced when both cell-cell contact and paracrine factors were present.

An important feature of hMSC is its alleged inhibitory effect on T cell proliferation (13, 34, 35). Our data showed that in PBMC co-cultures, proliferation of CD4^+^ T cells was not suppressed in either DCC or TW conditions. However, in purified CD4^+^ T cells, proliferation was clearly delayed in the direct cell contact situation. Blocking the metabolic activity and secretion precluded this effect. When paracrine factors were blocked in UC-MSC (BPL-treated UC-MSC) there was no suppression in the proliferation, this is in accordance with the literature (13) and also explains the stronger response when UC-MSC were co-cultured with only negatively selected CD4^+^ T cells. The reason for the lack of suppression in PBMC co-cultures might be the overstimulation by both αCD3/CD28 beads and surrounding cells in the PBMC mixture.

CD25 is a well-known marker of T cell activation, but is also highly expressed on regulatory T cells (36). While some studies suggested that hMSC suppress CD25 expression (17, 37), others showed an increase in the form of an induced Treg population (16, 38). With respect to the activation status, the addition of UC-MSC reduced CD25^+^ T cell population early on in the PBMC co-culture, but this was not observed when the cells were co-cultured with purified CD4^+^ T cells. This suggests that the reduction in CD25 expression might be mediated through other cells in the PBMC pool. In the other conditions (BPL-UC-MSC, TW, CM50% and CM25%), the activation marker expression was not as high and stable enough as in the direct cell contact culture condition with UC-MSC. These findings highlight that neither paracrine factors, nor cell-cell contact alone can regulate CD25 expression on T cells, as was the case when both factors were present at the same time. This suggests that regulation of the activation marker is a result of a dual mechanism. Regarding induction of T_reg_, we observed in the presence of either UC-MSC or BPL-UC-MSC, that a CD4^+^CD25^high^CD127^low^FOXP3^+^ population emerged. However, this population was not long-lived, even though there was sufficient IL-2 in the culture medium. Transient expression of FOXP3 in CD4^+^CD25^+^ T cells was previously shown to be a feature of early activation (39). This finding would support the presence of early activated T cells, rather than suppressive Treg in the UC-MSC co-cultures.

UC-MSC had a clear effect on CD4^+^ T cell differentiation. We found that UC-MSC suppressed T_EM_ and increased T_CM_ formation. This happened in both PBMC and pure CD4^+^ co-cultures, which shows the direct effect of UC-MSC on CD4^+^ T cells in inducing memory formation. Further, TW was not as efficient as DCC in suppressing T_EM_. Yet, the levels of T_CM_ cells were comparable until day 3 in both TW and DCC. T_EM_ and T_CM_ populations might therefore be differentially regulated by UC-MSC-specific factors and paracrine factors alone were not strong enough to downregulate T_EM_. BPL treatment of UC-MSC, by which paracrine factors are eliminated, did not lead to efficient reduction of T_EM_ or increase T_CM_ percentages in co-cultures, since there was a delay in occurrence of phenotypical changes. Together, these results imply that cell-cell contact alone is not strong enough to induce a phenotype shift in CD4^+^ T cells. Thus, the combination of both direct cell-cell contact and the presence of paracrine factors has the most outspoken effect in the induction of a T_CM_ population.

During acute or chronic inflammation, T cells are exposed to persistent antigenic/inflammatory signals. This directs T cells to be activated and differentiated into effector T cells. Following T cell expansion and resolution of inflammation, a subset of T cells die off, and the remainder become memory cells. However, in the case of constant exposure to signals, T cells might become hyporesponsive, and the inflammatory environment may give rise to exhaustion of T cells (40). Therefore, induction of T_CM_ upon UC-MSC encounter shows that UC-MSC induced a long-term protective T cell population, rather than an exhausted one. Notably, although a previous study (35) reported that hMSC were able to induce hyporesponsiveness in T cells, we found no evidence for this in our set up. When sorted T_CM_ were re-stimulated with αCD3/CD28 beads in the absence of UC-MSC, they proliferated and were skewed into a T_EM_ phenotype. This suggests that the inhibitory effect of the UC-MSC is transient and can be reversed upon a new encounter with the cognate antigen. This is of relevance for the clinical use of UC-MSC. UC-MSC do not render them anergic or exhausted, but rather drive them to a more mature state, whereafter T cells can respond to the same or different stimuli in case of (re-) encounter at later time point. Besides their protective role against antigens, these cells can migrate to secondary lymphoid organs to evoke immune response by B cells or CD8^+^ T cells to enhance their response or killing activity of infected cells [reviewed in (41)].

Among the measured anti- and pro-inflammatory cytokines, the levels of IL-6 and TGFβ were significantly affected by UC-MSC in co-cultures with CD4^+^ T cells. IL-6 was shown to influence the pluripotency and immune privileged status of the hMSC (42), whereby transcriptional blockade of IL-6 in hMSC inhibited their ability to reduce proliferation in a PBMC (43). Due to the pleiotropic nature of IL-6, its immunogenic potential is not well-defined, but it is known to function depending on environmental cues. IL-6 was able to orchestrate the expression of adhesion molecules on T cells by inducing L-selectin (44). This holds promise since the T_CM_ population is known for their expression of CCR7 and CD62L (L-Selectin) (45). Neutralizing IL-6 and TGFβ in co-cultures did not lead to a significant effect on memory formation, although especially in the case of IL-6, we found a clear trend towards a memory switch. Since, the BPL experiments indicated that paracrine factors are needed to execute a more rapid and elevated response and neutralization of IL-6 and TGFβ only revealed a trend, it can be envisaged that other factors such as extracellular vesicles or other cytokines/chemokines might play a role in UC-MSC function (46). However, contribution of specifically IL-6 should not be undervalued and its role should be further studied.

The difference in cytokine profile between direct cell contact and transwell conditions highlight the bidirectional communication between UC-MSC and CD4^+^ T cells. It is tempting to speculate that maintenance of CD25^+^ on activated T cells might be one of the mechanisms how UC-MSC act on CD4^+^ T cells. TGFβ was also shown to be an important mediator for T cell proliferation (47). Having increased levels of TGFβ in direct cell contact as compared to BPL-UC-MSC and the delay in proliferation of CD4^+^T cells, further supports a role of TGFβ in regulating proliferation by UC-MSC.

It was already shown that priming hMSC with pro-inflammatory cytokines, such as IL-1β, improves their immunoregulatory response (48). Moreover, a microenvironment that contains IFNγ (49) and IL-1β (48) was shown to be important for determining the immunomodulatory function of hMSC. Therefore, the presence of pro-inflammatory cytokines (such as IL-1β, IFNγ and IL-17 in co-cultures might be important for UC-MSC to exert their suppressive behavior (50).

**Figure 9:**
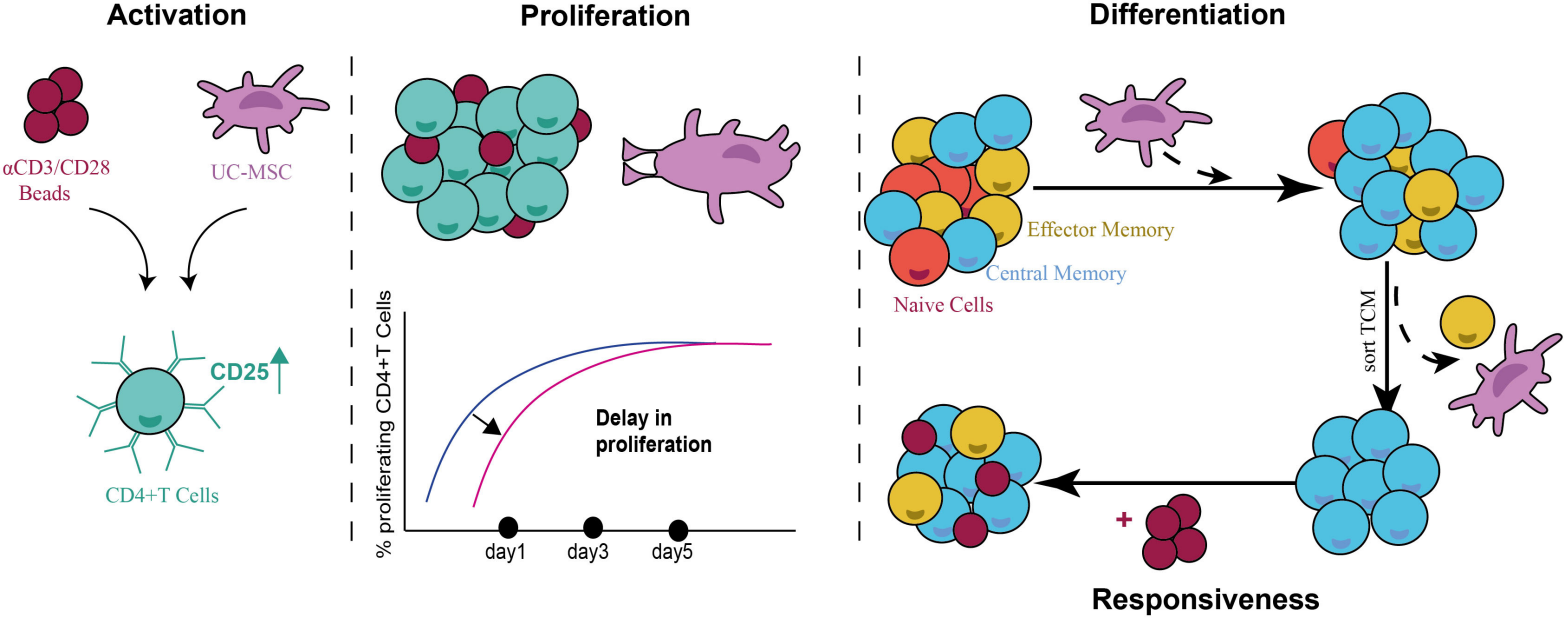
Schematic representation summarizing the immunomodulatory activity of UC-MSCs on CD4^+^ T cells. UC-MSC induce the expression of activation marker-CD25- on CD4^+^ T cells; while causing a delay in their proliferative state. UC-MSC induced a T_CM_ population in activated CD4^+^ T cells, and this phenotype was reversible when UC-MSC were removed from the co-culture and re-stimulated. This suggests a transient role of UC-MSC in modulating the immune response.

In conclusion, our results clearly show that UC-MSC induce an anti-inflammatory T cell phenotype by inducing a T_CM_ population in αCD3/CD28 beads activated CD4^+^ T cells. This activity was dependent on both cell-cell contact and paracrine factors, amongst which IL-6. Furthermore, the impact of UC-MSCs on CD4^+^ T cells was transient, since the responsiveness was restored upon restimulation (**Figure 9**).

## Data availability statement

The original contributions presented in the study are included in the article/**Supplementary Material**. Further inquiries can be directed to the corresponding author.

## Ethics statement

The studies involving human participants were reviewed and approved by Commissie Mensgebonden Onderzoek region Arnhem-Nijmegen. The participants provided their written informed consent to participate in this study. The patients/participants provided their written informed consent to participate in this study.

## Author contributions

ES, RM, MJ and IJ conceived the study. ES performed the experiments and data analysis. BC and ES were involved in data analysis an provided technical assistance. ES wrote the manuscript. RM, MJ, IJ, VB, DO and TW assisted on writing and editing the manuscript. All authors contributed to the article and approved the submitted version.

## Supplementary material

Supplementary Figure 1 Details of the experimental setup. Graphical overview of experimental set-up **(A)**. Metabolic activity, MTT (3-[4,5-dimethylthiazol-2-yl]-2,5- diphenyltetrazolium bromide), assay was performed to measure the activity of UC-MSCs after treatment with BPL **(B)**. n=6, and paired-t-test used to assess differences between each group; *p<0.05, **p<0.01, ***p<0.001.

Supplementary Figure 2 Phenotypical characterization of CD4^+^ T cells upon co-culture with UC-MSC or BPL treated UC-MSC. CD4^+^ T cells were co-cultured with UC-MSC (with or without BPL) after 24 hours of an αCD3/CD28 beads stimulation (CD4^+^+Beads+UC-MSC vs CD4^+^+Beads+BPL-UC-MSC). Phenotypical changes were monitored over 5-days by flow cytometry measurement. Frequencies of CD4^+^CD45RA^-^CCR7^-^ (T_EM_) **(A)**, CD4^+^CD45RA^-^CCR7^+^ (T_CM_) **(B)** were presented. Intracellular IFNγ **(C)** and IL-2 **(D)** levels were depicted on T_EM_ population. Reduced cytokine expression among T_EM_ and T_CM_ populations was represented as dot plots **(E)**. n=6, and paired-t-test used to assess differences between each group; *p<0.05, **p<0.01, ***p<0.001, ****p<0.0001.

Supplementary Table 1 List of monoclonal antibodies used in 4 panels.
