# Supplementary figures and images for: Umbilical cord-mesenchymal stem cells induce a memory phenotype in CD4+ T cells
Source: Front Immunol. 2023 Jun 20;14:1128359. doi: 10.3389/fimmu.2023.1128359 (PMC10318901; doi:10.3389/fimmu.2023.1128359)

A.

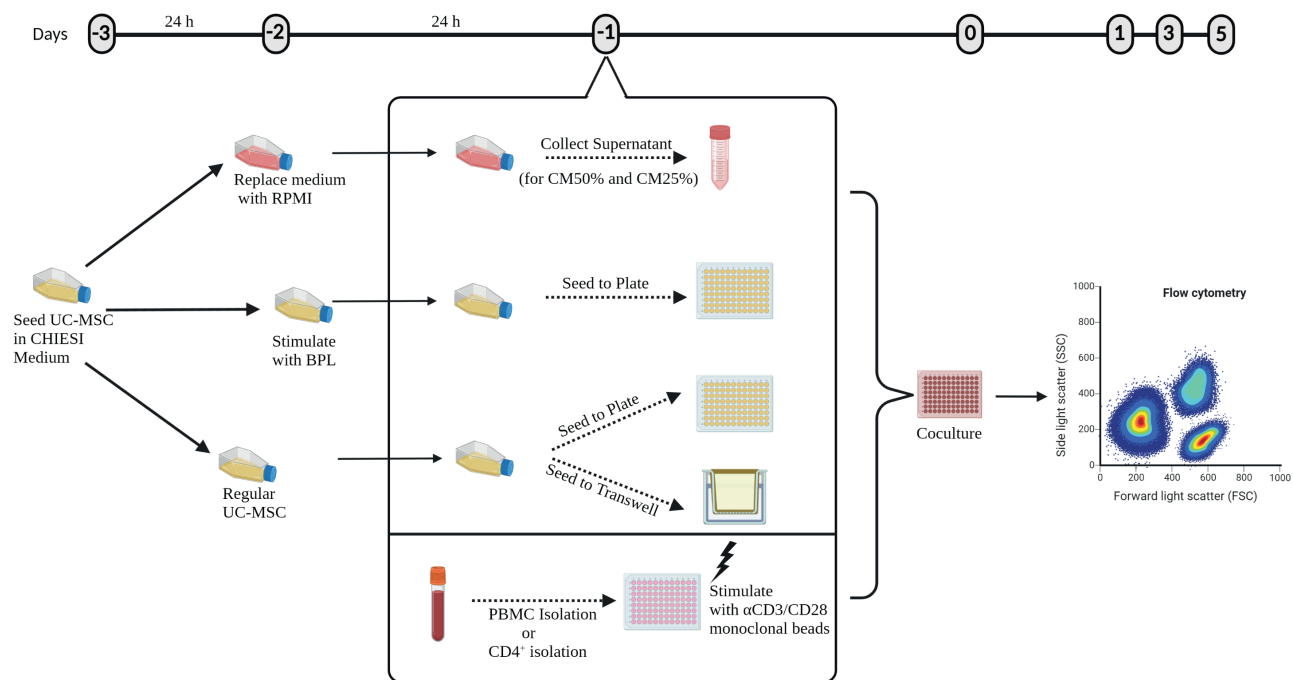

B.

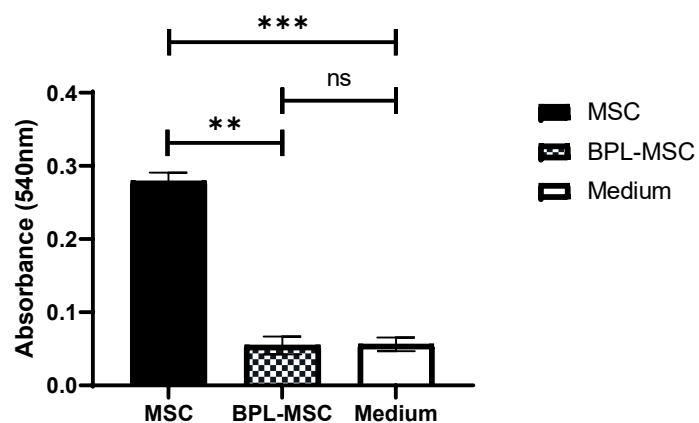

Supplement: Supplementary Figure 1 — Details of the experimental setup. Graphical overview of experimental set-up (A). Metabolic activity, MTT (3-[4,5-dimethylthiazol-2-yl]-2,5- diphenyltetrazolium bromide), assay was performed to measure the activity of UC-MSCs after treatment with BPL (B). n=6, and paired-t-test used to assess differences between each group; *p<0.05, **p<0.01, ***p<0.001. [file DataSheet_1.pdf]

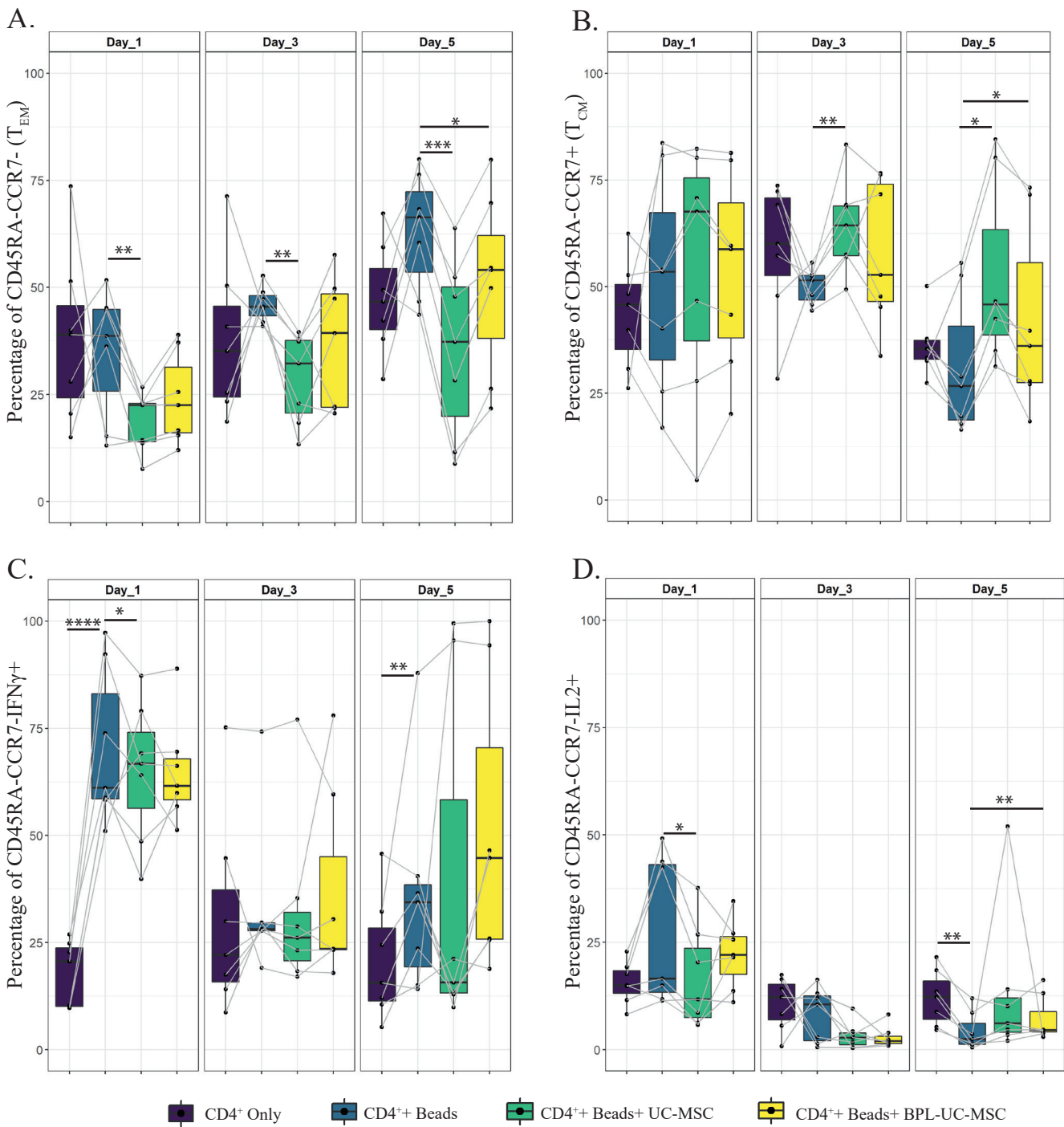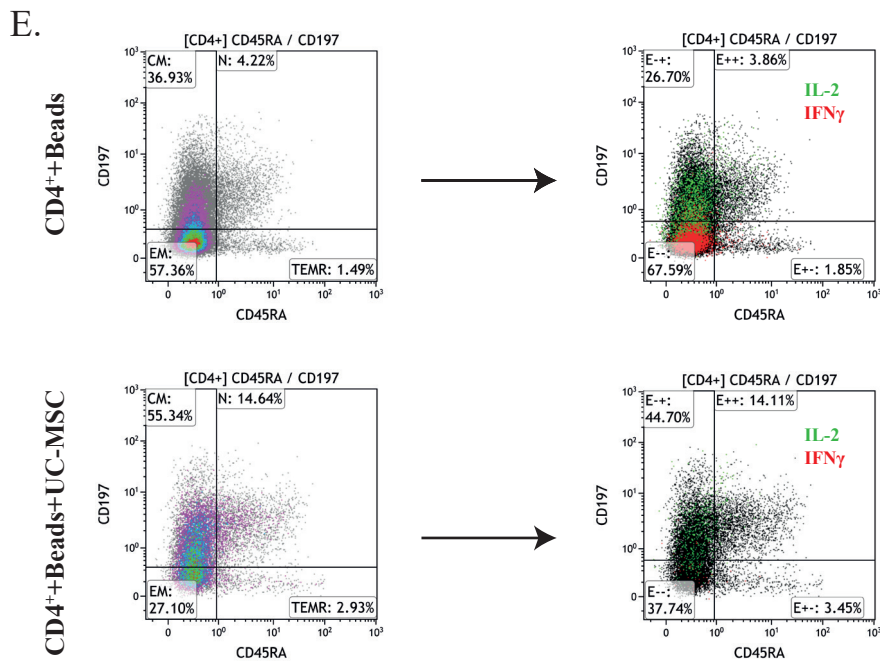

Supplement: Supplementary Figure 2 — Phenotypical characterization of CD4+ T cells upon co-culture with UC-MSC or BPL treated UC-MSC. CD4+ T cells were co-cultured with UC-MSC (with or without BPL) after 24 hours of an αCD3/CD28 beads stimulation (CD4++Beads+UC-MSC vs CD4++Beads+BPL-UC-MSC). Phenotypical changes were monitored over 5-days by flow cytometry measurement. Frequencies of CD4+CD45RA-CCR7- (TEM) (A), CD4+CD45RA-CCR7+ (TCM) (B) were presented. Intracellular IFNγ (C) and IL-2 (D) levels were depicted on TEM population. Reduced cytokine expression among TEM and TCM populations was represented as dot plots (E). n=6, and paired-t-test used to assess differences between each group; *p<0.05, **p<0.01, ***p<0.001, ****p<0.0001. [file DataSheet_2.pdf]
