# Supplementary material for: Umbilical cord-mesenchymal stem cells induce a memory phenotype in CD4+ T cells
Source: Front Immunol. 2023 Jun 20;14:1128359. doi: 10.3389/fimmu.2023.1128359 (PMC10318901; doi:10.3389/fimmu.2023.1128359)
Supplement: Supplementary Table 1 — List of monoclonal antibodies used in 4 panels. [file Table_1.pdf]

Supplementary Table 1. List of monoclonal antibodies used in 4 panels.

Antibody	Producer	Catalogue #
CD3-ECD	Beckman Coulter	A07748
CD4-PE Cy5.5	Beckman Coulter	B16491
CD8- PE Cy5	Beckman Coulter	A07758
CD11b- PE Dazzle 594	BioLegend	301348
CD14-ECD	Beckman Coulter	B29391
CD19-ECD	Beckman Coulter	A07770
CD25-PE Cy7	Beckman Coulter	557741
CD45- KrO	Beckman Coulter	B36294
CD45RA-FITC	Beckman Coulter	A07786
CD45RA-ECD	Beckman Coulter	B49193
CD56-ECD	Beckman Coulter	A82943
CD71-FITC	Life Technologies	MHCD7101
CD73-PE	BioLegend	344003
CD90- PerCp Cy5	BioLegend	328117
CD105- PE Cy7	Invitrogen	25-1057-42
CD127-APC AF700	Beckman Coulter	A71116
CD197-BV421	BioLegend	353208
HLA-DR- ECD	Beckman Coulter	IM3636
HLA-DR-PE	Beckman – Coulter	IM1639U
FOXP3-e450	eBioscience	48-4776-42
IL2-FITC	BD Biosciences	554565
IL2-PE	BD Biosciences	554566
IFN- AF700	BioLegend	506516
Ki67-AF647	BD Biosciences	558615
Ki67-AF488	BD Biosciences	561165
Fixation-Viability-APC e780	eBioscience	65-0865
